# Deletion of Tet proteins results in quantitative disparities during ESC differentiation partially attributable to alterations in gene expression

**DOI:** 10.1186/s12861-019-0196-6

**Published:** 2019-07-08

**Authors:** Michael Reimer, Kirthi Pulakanti, Linzheng Shi, Alex Abel, Mingyu Liang, Subramaniam Malarkannan, Sridhar Rao

**Affiliations:** 1 Blood Research Institute, Versiti, 8733 West Watertown Plank Road, Milwaukee, WI 53226 USA; 2 Vanderbilt University, Nashville, TN 37240 USA; 3 Department of Microbiology and Immunology, Medical College of Wisconsin, Milwaukee, WI 53226 USA; 4 Department of Physiology, Center of Systems Molecular Medicine, Medical College of Wisconsin, Milwaukee, WI 53226 USA; 5 Department of Medicine, Medical College of Wisconsin, Milwaukee, WI 53226 USA; 6 Department of Cell Biology, Neurobiology, and Anatomy, Medical College of Wisconsin, Milwaukee, WI 53226 USA; 7 Department of Pediatrics, Medical College of Wisconsin, Milwaukee, WI 53226 USA

**Keywords:** Embryonic stem cells, DNA methylation, Ten eleven translocation (TET proteins), Differentiation

## Abstract

**Background:**

The Tet protein family (Tet1, Tet2, and Tet3) regulate DNA methylation through conversion of 5-methylcytosine to 5-hydroxymethylcytosine which can ultimately result in DNA demethylation and play a critical role during early mammalian development and pluripotency. While multiple groups have generated knockouts combining loss of different Tet proteins in murine embryonic stem cells (ESCs), differences in genetic background and approaches has made it difficult to directly compare results and discern the direct mechanism by which Tet proteins regulate the transcriptome. To address this concern, we utilized genomic editing in an isogenic pluripotent background which permitted a quantitative, flow-cytometry based measurement of pluripotency in combination with genome-wide assessment of gene expression and DNA methylation changes. Our ultimate goal was to generate a resource of large-scale datasets to permit hypothesis-generating experiments.

**Results:**

We demonstrate a quantitative disparity in the differentiation ability among Tet protein deletions, with *Tet2* single knockout exhibiting the most severe defect, while loss of *Tet1* alone or combinations of *Tet* genes showed a quantitatively intermediate phenotype. Using a combination of transcriptomic and epigenomic approaches we demonstrate an increase in DNA hypermethylation and a divergence of transcriptional profiles in pluripotency among Tet deletions, with loss of *Tet2* having the most profound effect in undifferentiated ESCs.

**Conclusions:**

We conclude that loss of *Tet2* has the most dramatic effect both on the phenotype of ESCs and the transcriptome compared to other genotypes. While loss of Tet proteins increased DNA hypermethylation, especially in gene promoters, these changes in DNA methylation did not correlate with gene expression changes. Thus, while loss of different *Tet* proteins alters DNA methylation, this change does not appear to be directly responsible for transcriptome changes. Thus, loss of Tet proteins likely regulates the transcriptome epigenetically both through altering 5mC but also through additional mechanisms. Nonetheless, the transcriptome changes in pluripotent *Tet2*^*−/−*^ ESCs compared to wild-type implies that the disparities in differentiation can be partially attributed to baseline alterations in gene expression.

**Electronic supplementary material:**

The online version of this article (10.1186/s12861-019-0196-6) contains supplementary material, which is available to authorized users.

## Background

DNA methylation plays a critical role in regulating gene expression during development and is maintained in mammals through a complex interplay between DNA methyltransferases (DNMT) and a family of proteins termed Ten Eleven Translocation (Tet). DNMTs are the “writer” of DNA methylation, whereas Tet proteins are the “erasers” [1–4]. All Tet members are capable of oxidizing methylated cytosines (5mC) to 5-hydroxymethylated cytosine (5hmC). Further oxidation of the 5hmC mark by Tet proteins yields 5-carboxy cytosine (5caC) and 5-formyl cytosine (5fC; [5]) which are unstable and removed by base excision repair (BER; [6–8]), resulting in conversion to an unmodified cytosine and is referred to as active DNA demethylation. Tet proteins can also cause DNA demethylation through a passive mechanism. The 5hmC mark is not recognized by maintenance DNMTs during DNA replication and will be absent in the daughter strand, resulting in passive DNA demethylation in a cell-cycle dependent manner. Regulation of DNA methylation is especially important during early embryogenesis where dynamic, rapid changes in the epigenome are required for early differentiation steps.

Tet1 and Tet2 are highly expressed in the inner cell mass (ICM; [9]) from which murine embryonic stem cells (ESCs) are derived, but studies in ESCs on the role of Tet proteins have resulted in conflicting reports. In vivo studies of Tet deletion have established that loss of either *Tet1* [10] or *Tet2* [11] alone had no effect on early development. The combination deletion of *Tet1* and *Tet2* (double knockout, DKO) exhibited a partially penetrant phenotype in which a fraction of embryos died perinatally, but others were overtly normal [12]. Even though *Tet3* is only expressed at low levels in ESCs and early embryos, combined loss of *Tet1*, *Tet2*, and *Tet3* in ESCs prevents them from contributing to chimeric embryos during blastocyst complementation [13], implying that some amount of Tet protein activity is required for normal embryonic development. Importantly, because of the complex cell:cell interactions within the developing embryo, as well as differences in mouse strain background, it has not been possible to translate these in vivo results into how Tet proteins regulate ESCs and pluripotency. There remains debate about which *Tet* genes are required for pluripotency and/or differentiation, and how they may interact in combination remains unclear. Part of this likely relates to the differences in how the ESC were isolated, differences between ESC genetic background, as well as the use of classical homologous recombination approaches which induce large genomic deletions [10, 12, 13]. Genomic editing, which can generate null alleles by inducing small frame-shifts to prevent functional protein production rather than large genomic deletions, has been used at all the *Tet* genes [14, 15], but the resulting ESCs have not been carefully quantitatively characterized in terms of their differentiation and pluripotency. To address this, we utilized a GFP reporter line to permit quantitative tracking of pluripotency in combination with combinatorial genomic-editing to ablate all three *Tet* genes in an isogenic background. By utilizing both transcriptome and genome-wide DNA methylation analysis we establish that the differences we observe are at least partially related to baseline differences in the epigenome and transcriptome of these cells.

## Results

### Deletion of Tet proteins does not disrupt pluripotency

**Fig. 1:**
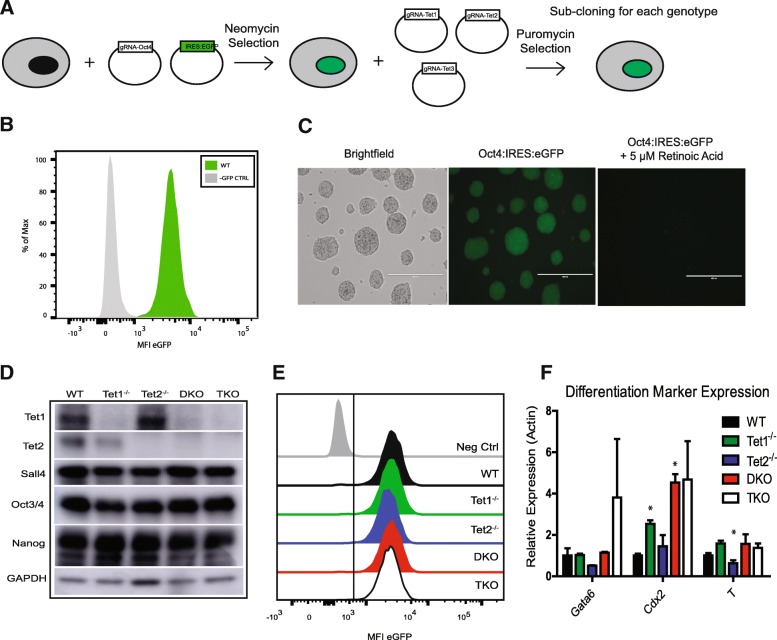
Loss of Tet proteins does not impair pluripotency. **a** Schematic showing the generation of the Oct4:IRES:eGFP parental cell line followed by deletion of each TET protein individually or combinatorially (DKO = *Tet1*^−/−^:*Tet2*^−/−^, TKO = *Tet1*^−/−^:*Tet2*^−/−^:*Tet3*^−/−^). **b** Representative histogram showing eGFP expression in a WT clone. Parental (GFP-) mESC was used as a negative control. **c** Brightfield and fluorescent micrographs (20x) of WT Oct4:IRES:eGFP before and after exposure to 5 μM retinoic acid to induce differentiation. **d** Representative western blot of Tet1,Tet2, and common pluripotency factors in each line. GAPDH was used as a loading control. **e** Representative histogram of eGFP expression in WT, *Tet1*^−/−^, *Tet2*^−/−^, DKO, and TKO clone. **f** RT-qPCR of endoderm (Gata6), trophectoderm (Cdx2), and mesoderm (Brachyury/T) associated transcription factors. * = *p* < .05 compared to WT.

We utilized a previously generated ESC line in which eGFP was “knocked-in” the *Pou5f1* locus, permitting use of eGFP expression as a quantitative marker of pluripotency (Figs. 1a-c). A single clone was used as a parental line for all further experiments and hereafter referred to as wild-type (WT). Published gRNAs [14] to all three *Tet* genes were transfected into the parental line to generate the following lines: *Tet1*^*−/−*^*, Tet2*^*−/−*^*,* DKO (*Tet1*^*−/−*^*:Tet2*^*−/−*^*),* and TKO (*Tet1*^*−/−*^*:Tet2*^*−/−*^*:Tet3*^*−/−*^; Fig. 1a). Unless otherwise noted, all experiments were performed with 3 individual clones of WT, *Tet1*^−/−^ and 2 clones of *Tet2*^−/−^, DKO, TKO. Generation of indels by genomic editing was confirmed by direct sequencing (Additional file 4: Table S4) and loss of protein by Western blot (Fig. 1d). All Tet deletion lines expressed similar levels of eGFP (Fig. 1e) and common pluripotency markers (Fig. 1d). Expression of three lineage markers, Gata6 (endoderm), Cdx2 (trophectoderm), and Brachyury (mesoderm) were variable across lines (Fig. 1f) when measured by RT-qPCR, likely due to clone-to-clone variation given the very low-level expression of these markers. These data are consistent with published reports that deletion of Tet proteins in any combination does not alter pluripotency per se.

### Deletion of Tet proteins impairs differentiation

**Fig. 2:**
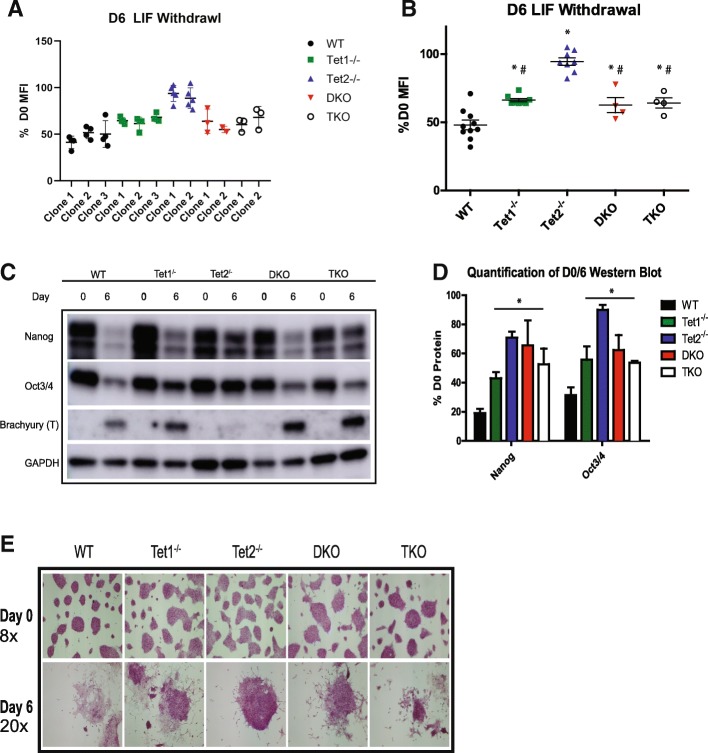
Loss of Tet proteins impairs differentiation. **a** Column scatter plot showing the ratio of D6 Mean Fluorescence Intensity (MFI) to D0 eGFP MFI of each cell line. Individual clones are shown to demonstrate minimal inter-clone variability. **b** Similar to (A), but clones are grouped together for the purpose of statistical tests. * = *p* < .05 as compared to WT, # = p < .05 compared to Tet2−/−. WT *n* = 10, Tet1−/− *n* = 8, Tet2−/−n = 8, DKO *n* = 4, TKO *n* = 4. **c** Representative western blot of the pluripotency factors Nanog, Oct3/4 and the mesoderm marker Brachyury (T). **d** Quantification of western blots for Nanog and Oct3/4. *n* = 2. * = *p* < .05, compared to WT. **e** Brightfield micrographs (top panel) of alkaline phosphatase staining at day 0 (8x) and following 6 days (bottom panel) of LIF withdrawal (20x)

To determine the effect of Tet deletion on differentiation we performed a leukemia inhibitory factor (LIF) withdrawal assay. LIF maintains ESC pluripotency and its withdrawal promotes differentiation down all three germ layers. All lines were grown without LIF for 6 days and differentiation assayed by flow cytometry for eGFP expression. All Tet deletion lines, including independent clones for each genotype, were resistant to differentiation as measured by retention of eGFP expression, consistent with prior publications (Fig. 2a, b; [10, 12, 13, 16]). *Tet2*^*−/*^ clones^*−*^, as expected, were quantitatively more resistant to differentiation as expected than the other lines [17]. Surprisingly, the DKO and TKO lines more closely resembled the *Tet1*^*−/*^ lines^*−*^, which displayed an intermediate phenotype. This suggests that *Tet1* deletion is the “dominant” phenotype since the DKO and TKO differentiation mirrors *Tet1*^−/−^ and not *Tet2*^−/−^. Given the agreement among clones, we used multiple clones for all subsequent analyses and grouped them as biological replicates, thereby allowing interclonal variations to drive our statistical tests.

To confirm the loss of pluripotency and resistance to differentiation using an independent approach, we performed Western blots for the pluripotency factors Nanog and Oct3/4 and the mesoderm marker Brachyury/T (Fig. 2c). All Tet genotypes retained higher levels of both Nanog and Oct3/4 when compared to the WT (Fig. 2c). Importantly, Oct3/4 protein levels as measured by Western blot closely resemble the eGFP expression, indicating our flow cytometry approach is a quantitative measurement of pluripotency. To identify morphological differences during differentiation, cells were stained for alkaline phosphatase (AP) at day 0 and 6 of LIF withdrawal (Fig. 2e). At D0 no gross morphological changes were observed in any Tet deletion lines (Fig. 2e, top panel). Following 6 days of LIF withdrawal, WT colonies at D6 appear flatter and are negative for AP, *Tet2*^−/−^ colonies were less spread out than WT and remained AP positive whereas the other lines displayed an intermediate phenotype (Fig. 2e, bottom panel). In agreement with several published studies, we found no defects in pluripotency following deletion of Tet proteins but uncovered a quantitative discrepancy between *Tet2*^*−/−*^ and other genotypes which phenocopied *Tet1*^*−/−*^. This is in contrast to previous studies that suggest TKO cells display the most severe differentiation block [10, 12, 13, 16]. Importantly, because we did not continue our experiments past D6, we cannot formally distinguish between a true “block” in differentiation or simply a “delay” in differentiation. Distinguishing between the two possibilities would require a longer time course to determine if the *Tet2*^*−/−*^ cells eventually differentiate completely. Overall, our use of an isogenic parental line and multiple clones demonstrated a unique quantitative disparity among Tet proteins during ESC differentiation.

### Transcriptional profiling of Tet deletions in pluripotency and LIF withdrawal

**Fig. 3:**
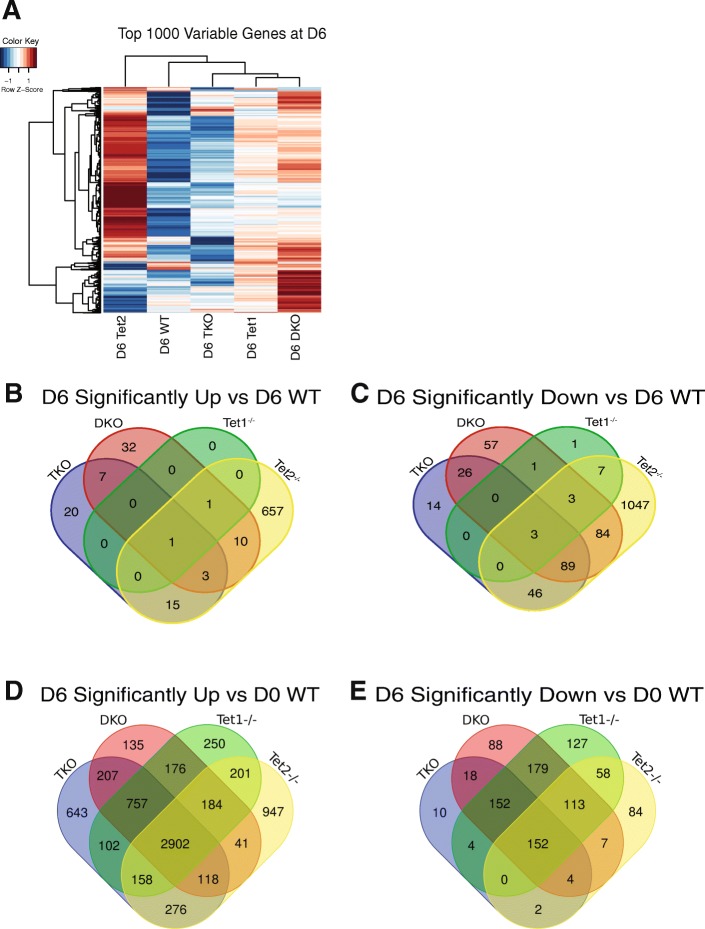
RNA-seq analysis of D6 LIF withdrawal samples. **a** Heatmap of top 1000 most variable genes at D6. **b** Venn diagram of significantly Up (p < .05, |log2 fold change| > 1) comparing different knockouts to D6 WT cells. **c** Venn diagram of significantly Up (p < .05, |log2 fold change| > 1) comparing different to D6 WT cells. **d** Similar to (B) but compared to D0 WT cells. **e** Similar to (C) but compared to D0 WT cells

To delineate the disparities in differentiation among the various genotypes, RNA-seq was performed at D6 of LIF withdrawal. We chose LIF withdrawal because it permits ESCs to differentiate down multiple lineages through loss of a signaling cascade rather than a strong positive differentiation signal which may promote its own set of transcriptome changes. We performed RNA sequencing (RNA-seq) on differentiated (D6) samples and read counts were normalized using ERCC spike-ins to enhance quantification (Additional file 2: Table S2, [18]). D6 analysis of the top 1000 most variable genes across genotypes revealed a unique transcriptional profile of *Tet2*^−/−^ cells that was not shared by other Tet deletions or the WT based upon unsupervised hierarchical clustering, consistent with the *Tet2*^*−/−*^ cells exhibiting a very different cell state after LIF withdrawal compared with the other genotypes (Fig. 3a). Consistent with our eGFP analysis, the *Tet1*^*−/−*^, DKO, and TKO cells most closely resembled each other at the transcriptome level, again indicating that the *Tet1*^*−/−*^ intermediate differentiation phenotype was dominant over *Tet2*^*−/−*^*.* To further refine which genes were differentially expressed, we utilized both a statistical (adjusted p-val < 0.05) and fold change (> 2-fold) criteria to generate refined gene lists which likely represented the most differentially expressed as compared to WT cells differentiated in parallel. In terms of the genes which were underexpressed (Fig. 3b) or overexpressed (Fig. 3c) we found that the *Tet2*^*−/−*^ cells exhibited the most uniquely dysregulated genes compared to WT, consistent with *Tet2*^*−/−*^ being in a distinct cell state compared to differentiated WT cells. Our results are consistent with the Tet2^*−/−*^ cells at D6 of differentiation being very different from WT, with *Tet1*^*−/−*^, DKO, and TKO cells representing an intermediate defect in differentiation following LIF withdrawal. To compare grossly the gene expression changes when compared to undifferentiated (D0) WT cells, we performed a similar analysis (Fig. 3d,e). Not surprisingly, all the genotypes showed substantially more changes in gene expression in terms of upregulated genes. This is consistent with the different genotypes, including *Tet2*^*−/−*^ showing some amount of differentiation. However, in general, there was far more overlap with among the genotypes with the exception of *Tet2*^*−/−*^, consistent with its unique phenotype.

**Fig. 4:**
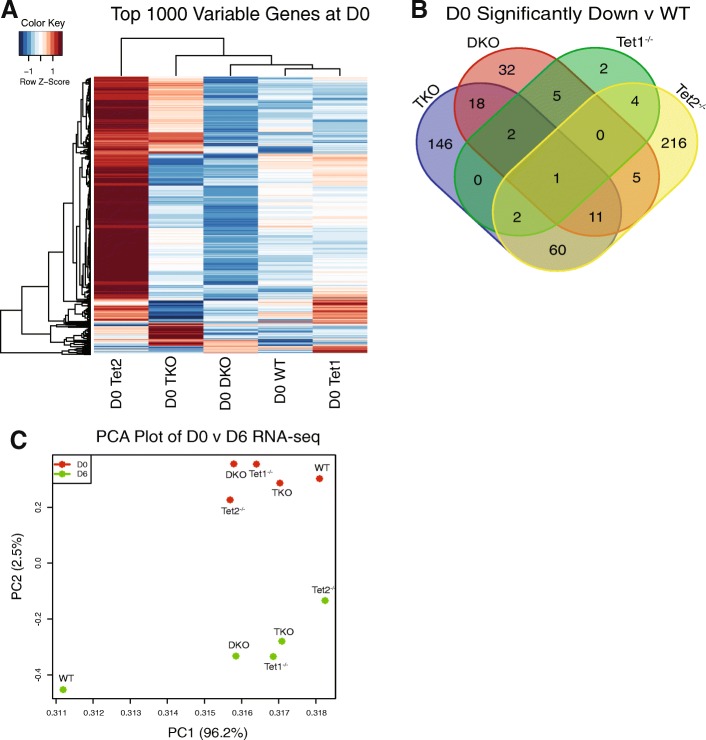
RNA-seq analysis following D0. **a** Heatmap of top 1000 variable genes at D0. **b** Venn diagram of significantly down (*p* < .05, log2 fold change <− 1) genes at D0, as compared to WT. **c** PCA plot of RNA-seq data from D0 (red) and D6 (green) samples. % variability encompassed within each principle components are shown in parentheses

Given our results, we wondered whether the loss of *Tet* genes induced baseline transcriptome changes in the ESCs which were compatible with pluripotency, or alternatively Tet proteins were only required during differentiation as posited in [17]. We performed RNA-seq on undifferentiated ESCs (D0) using the same approach as above. The top 1000 most variable genes are displayed as a heat map in Fig. 4a and indicate that *Tet2*^*−/−*^ cells are transcriptionally distinct from the other genotypes at D0. In contrast to the D6 results, the TKO cells appear to be distinct at D0 than the *Tet1*^*−/−*^, WT, and DKO cells. This was surprising, since *Tet3* is generally expressed at low levels in ESCs, although other groups have shown that *Tet3* expression is upregulated in DKO cells with minimal effects on global levels of 5hmC [12]. Nonetheless, our results are consistent with the *Tet2*^*−/−*^ having a very different gene expression pattern compared to the other genotypes. Given the role of Tet proteins in DNA demethylation, it was not surprising that compared to WT cells at D0 our differential expression analysis showed virtually all genes were downregulated (Fig. 4a). Not surprisingly, genes expressed at lower levels in *Tet2*^*−/−*^ versus WT cells were predominantly unique to this genotype and not shared with the others, again indicating that loss of *Tet2* induced a unique change on the transcriptome in ESCs compared to the other genotypes at D0.

Prior work from others has demonstrated that loss of *Tet2* in ESCs delays differentiation because of a failure to activate enhancers critical to differentiation programs [17]. Thus, loss of Tet proteins could prevent proper differentiation either through baseline transcriptome differences, an inability to activate differentiation-critical gene expression programs, or perhaps both. To address this, gene lists for significantly downregulated genes at D0 were compared for overlap between different knockouts (Fig. 4b). *Tet2*^−/−^ had the most downregulated genes (299) as compared to the WT and showed little overlap with any other Tet genotype besides TKO (60 shared). Taken together, we conclude that Tet proteins predominantly function to activate gene expression and *Tet*2^−/−^ induces unique transcriptome changes not shared by other Tet deletions at D0. This would be consistent with baseline transcriptome differences in the different genotypes at D0. Collectively, our data along with the literature [17] would suggest loss of Tet proteins prevents differentiation through multiple mechanisms.

To obtain a more global view of transcriptome differences between the various genotypes at both D0 and D6, we performed a principal component analysis (PCA; Fig. 4c). Consistent with our prior analysis, differentiated (D6) WT cells segregated into a unique quadrant, indicating they were highly distinct from the other cells. D6 DKO, TKO, and *Tet1*^*−/−*^ clustered together and were distinct from the D6 *Tet2*^*−/−*^, again confirming, using an alternative analytic approach, that D6 *Tet2*^*−/−*^ cells were different than other genotypes. Surprisingly, the D0 cells, all of which display a pluripotent phenotype, were less clustered than anticipated. The D0 WT cells were the most distinct, and again the D0 DKO, TKO, and *Tet1*^*−/−*^ formed a cluster. The D0 *Tet2*^*−/−*^ cells were distinct from the other cells again, indicating that at baseline their transcriptome is distinct from the other genotypes.

**Fig. 5:**
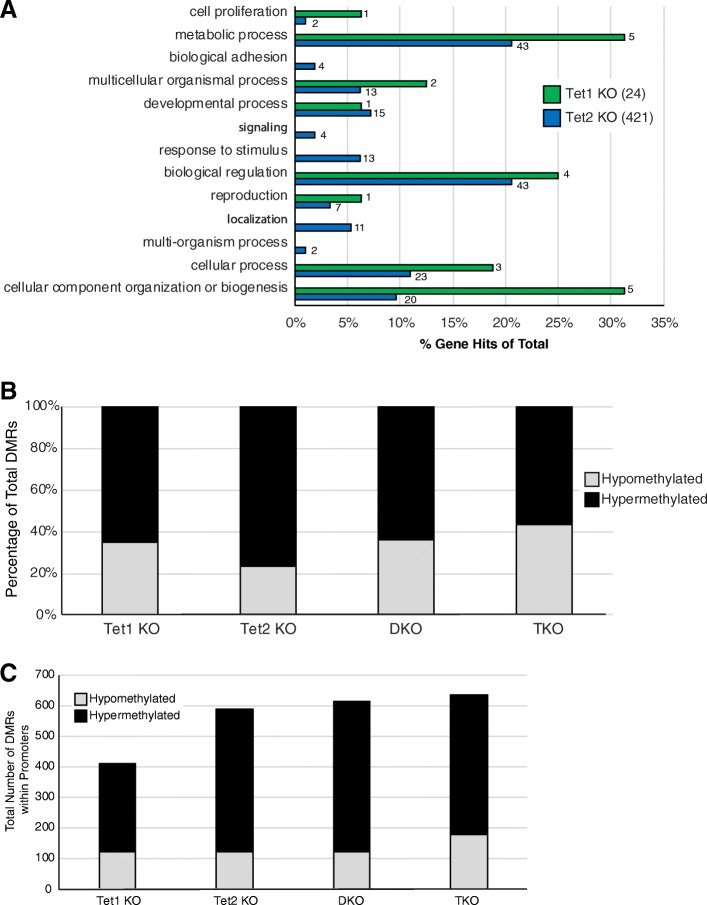
Gene ontology for altered gene expression and DNA methylation. **a** Gene Ontology terms for *Tet1*^*−/−*^ and *Tet2*^*−/−*^ down regulated genes compared to WT were identified using Panther. # to the right of each bar represents the number of downregulated genes identified in each pathway. The total number of genes used for each knock-out is shown in parentheses within the legend. **b** Percentage of hyper and hypomethylated DMRs as compared to WT. **c** Number of hyper and hypomethylated DMRs located in proximity to gene promoter/TSS

To better delineate the pathways which may be altered in *Tet1*^*−/−*^ and *Tet2*^*−/−*^ as compared to WT cells we first performed Gene Ontology analysis using PANTHER [19] to assign differentially expressed down-regulated transcripts to their respective biological process (Fig. 5a). Because *Tet1*^*−/−*^ had far fewer down-regulated transcripts (24) then *Tet2*^*−/−*^ (421), fewer overall pathways were identified overall. Importantly, there was rough agreement in general between the processes effected by loss of either *Tet1* or *Tet2*. Thus, while loss of *Tet2* had a more dramatic effect on the overall transcriptome then loss of *Tet1*, there was general agreement between the biological processes impacted by loss of either *Tet1* or *Tet2*.

### Tet deletion alters DNA methylation which does not correlate with gene expression changes

We next hypothesized that changes in DNA methylation in pluripotent ESCs explains their transcriptome differences, at least partially. To quantitate DNA methylation differences, we performed reduced representation bisulfite sequencing (RRBS) on D0 samples. RRBS is highly quantitative and provides robust coverage of CpG islands which are abundant in mammalian gene promoters (Additional file 3: Table S3). Differentially methylated regions (DMRs) were defined as having a change of at least 25% compared to WT. Overall, we observed more hypermethylated than hypomethylated regions in all Tet genotypes, consistent with their role in DNA demethylation (Fig. 5b; [3, 4]). It should be noted that the *Tet2*^*−/−*^ cells displayed the most hypermethylation overall compared to wild-type, whereas the *Tet1*^*−/−*^, DKO, and TKO all showed a similar change in hypermethylation. DMRs were found at consistent ratios in all genotypes across promoters, introns, exons, and intergenic elements (data not shown), consistent with a genome-wide change in DNA methylation. Importantly, when we looked at a small window around TSS (+/− 2 kb) corresponding to gene promoters, we observed a larger predominance of DNA hypermethylation rather than hypomethylation (Fig. 5c), consistent with prior literature that Tet proteins bind to a large fraction of promoters [20, 21].

**Fig. 6:**
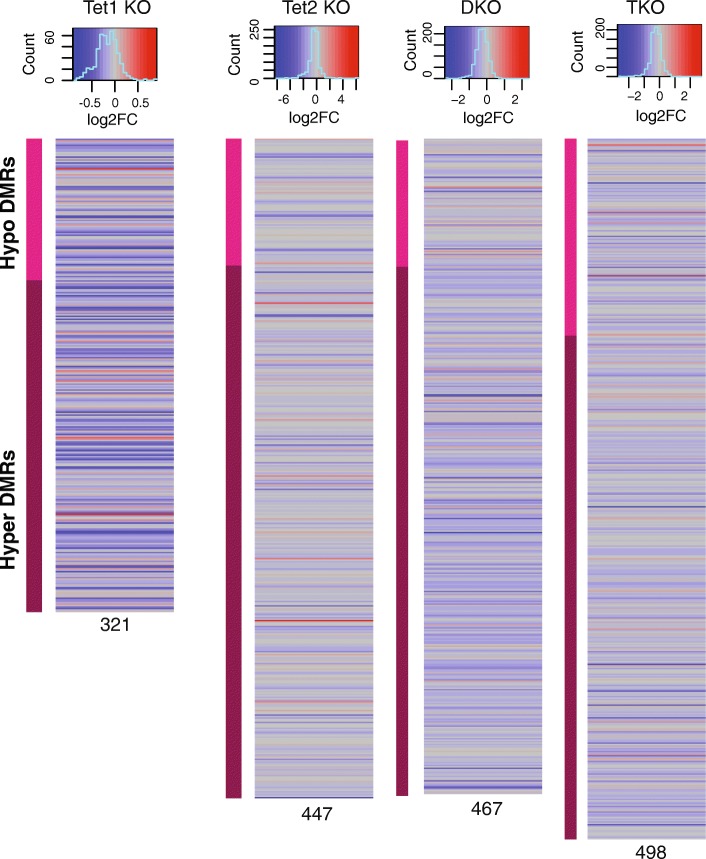
DNA methylation changes do not necessarily correlate with changes in gene expression. For each genotype (Tet1 KO, Tet2 KO, DKO, TKO), DMRs were identified in promoters as compared to WT. The corresponding Log2 Fold change in the gene’s expression was then graphed. Because the dynamic range across the different genotypes was so different, color schemes are different for each genotype and are shown at the top of each column. Total number of DMRs used for each genotype is shown below each column

To determine if changes in DNA methylation correlated with changes in mRNA, we identified DMRs within promoters for each genotype and then mapped the fold change in the gene (Fig. 6). Surprisingly, changes in DNA methylation at gene promoters did not correlate with transcriptional changes in the D0 RNA-seq. This is consistent with prior literature [10, 12, 13, 20, 21]. Thus, even though we utilized genomic editing in an isogenic background with multiple clones, we were unable to mechanistically link changes in DNA methylation to altered gene expression.

## Discussion

In this study we used CRISPR/Cas9 to generate *Tet1*^−/−^, *Tet2*^−/−^, DKO, and TKO in an isogenic ESC line. In accordance with published studies, we did not observe any perturbation of pluripotency following Tet deletion. We did however uncover a quantitative disparity across genotypes during differentiation. All genotypes were resistant to differentiation, consistent with published reports [10, 12, 13, 16]. Importantly, these experiments revealed three key findings. First, while loss of Tet proteins caused a block in differentiation, the loss of *Tet2* induced a near complete block with a > 90% retention of eGFP expression. Second, loss of *Tet1* caused only a partial reduction in differentiation. Third, is that in both DKO and TKO cells their differentiation potential phenocopied loss of *Tet1* rather than loss of *Tet2*, implying that the *Tet1*^*−/−*^ phenotype is dominant. This quantitative difference has not previously been described within the literature and was most likely difficult to conclusively observe because of the lack of isogenic backgrounds and our use of at least two, independent clones for all genotypes minimizes the chance that differences are simply related to clone-to-clone variation. In addition, by using LIF withdrawal rather than a potent differentiation agent such as retinoic acid we were able to observe a more subtle but nonetheless quantitative difference among genotypes. Importantly, many labs have published using TKO embryonic cells representing the most dramatic phenotype [13, 22]. Our data would argue this may not be the case, since the *Tet2*^*−/−*^ ESCs exhibit a substantial, quantitative reduction in their ability to differentiate.

Importantly, while our study focused on the role of *Tet* proteins in vitro, these findings are inconsistent with the observed in vivo phenotypes. As mentioned earlier, loss of either *Tet1* or *Tet2* appears to be well-tolerated by embryos, and in fact double knockouts (*Tet1*^*−/−*^*;Tet2*^*−/−*^) are able to generate healthy adults, although not at the expected Mendelian ratios [10–12]. Only triple knock-out (*Tet1*^*−/−*^*;Tet2*^*−/−*^;*Tet3*^*−/−*^) animals exhibit early developmental defects, illustrating that the in vivo and in vitro results of ESCs are not fully compatible [13]. Given the complex environment in vivo where both external signals including cell-cell interactions and diffusible signaling factors play critical roles in organismal development, it is perhaps not surprising there is discordance between our in vitro results and in vivo studies. Nonetheless, while our studies may not lend insights into the in vivo role of Tet proteins, they do provide some key insights into how they operate to regulate the transcriptome of pluripotent cells.

A second key area of disparity with the literature surrounds seminal studies by Meelad Dawlaty and Rudy Jaenisch [10, 12, 13], in which ESCs deficient in different combinations of Tet proteins were derived. In these studies, classical homologous recombination was used to generate animals with combinatorial loss of *Tet*s, and then ESCs were derived from blastocysts. While in vivo these cells contributed poorly to chimeras during blastocyst complementation, they were able to form embryoid bodies in vitro, consistent with our findings that TKO cells have an impaired but not complete block in differentiation. This disparity between our groups results and the Jaenisch findings could be for two different reasons. The first may be technical, given the significant differences between genomic editing in a single, parental ESC line as opposed to classical homologous recombination, breeding adult animals, followed by derivation of ESCs. The second is our use of an *Oct4-EGFP* reporter, originally developed by the Jaenisch group [15, 23], which permits quantitative measurement of pluripotency and a direct comparison among the different genotypes. Irrespective of the differences, it is clear from our work and others [17] that loss of *Tet2* has the most dramatic phenotype on ESC differentiation, but further studies will be required to explain the discordance between our work and the studies from others.

The second part of this study used genome-wide approaches to quantitate transcriptome and epigenomic differences. To our surprise given their phenotypic similarity at D0, RNA-seq showed substantial gene expression differences between *Tet2*^−/−^ and other genotypes. These changes did not affect pluripotency but imply the differences during LIF withdrawal are at least partly attributable to baseline disparities in the transcriptomes between genotypes. Importantly, we cannot exclude that Tet2 is also required for remodeling chromatin during differentiation [17], which warrants further investigation to tease apart these possibilities. From the D0 results we conclude that *Tet2*^*−*/−^ cells induce a unique transcriptional program as compared to loss of the other Tets. Given the profound block in differentiation we saw in the *Tet2*^*−/−*^ ESCs, we were surprised to find that additional deletion of *Tet1*^*−/−*^ in DKO or TKO cells more closely phenocopied *Tet1*^*−/−*^ cells, both in terms of their differentiation potential and their baseline transcriptome. This phenotype requires further investigation, since in terms of baseline transcriptome changes loss of *Tet1* alone had modest effects. This implies that the combinatorial loss of both *Tet1* and *Tet2* that is critical to the intermediate phenotype. Importantly, given the close correlation between the baseline D0 transcriptome of *Tet1*^*−/−*^, DKO, and TKO cells, it is likely that the intermediate differentiation phenotype again is at least partially attributable to the role of different Tet proteins in chromatin remodeling during differentiation. Nonetheless, this pattern of differentiation block among the various genotypes could only have been uncovered using a single, parental, isogenic line which easily permitted the quantitative measurement of pluripotency.

Since Tet proteins canonically function in DNA demethylation, we hypothesized that changes in D0 RNA-seq would be secondary to alterations in DNA methylation. Overall, we observed a majority of DMRs in the Tet deletion genotypes were hypermethylated compared to WT. *Tet2*^−/−^ had the largest number of hypermethylated DMRs even though this genotype had the second fewest of total DMRs. Nonetheless, our data is consistent with the work of others, in that there was little correlation between the changes in DNA methylation and transcriptome changes we observed [9, 10, 12, 13, 20]. This would imply that the observed transcriptome changes are separate from the role of Tet proteins in active DNA demethylation. It may be that our transcriptome differences are secondary to loss of 5hmC, which we did not directly measure.

Alternatively, the lack of differences between DNA methylation and transcriptome changes may be secondary to the dynamic interplay at CpG islands of DNA methylation and other, histone-based epigenetic marks. For example, CpG islands can become resistant to gaining DNA methylation by the presence of trimethylation of lysine 4 on histone 3 (H3K4me3; [24]). This may represent a potential mechanism to “bypass” the loss of active DNA demethylation at actively expressed genes marked by H3K4me3. In addition, Tet1 in particular forms protein:protein interactions with other epigenetic complexes, adding an additional layer of potential regulation. For example, Tet1 directly interacts with Sin3a [25], a core component of the repressive histone-deacetylase complex. Genes co-occupied by both Tet1 and Sin3a, such as *Lefty1* appear to be activated by the presence of both proteins, indicating that the combinatorial interaction of different epigenetic complexes must be examined in combination to truly appreciate their effects on gene expression. Another example includes so-called bivalent-marked genes, those with both the activating H3K4me3 and the Polycomb Repressive Complex 2 (PRC2) associated-mark H3K27me3. Multiple reports [21, 26–28] indicate that Tet1 can be recruited to these locations to modulate gene expression through a protein:protein interaction, which may or may not ultimately modulate DNA methylation within these promoters. This would further suggest that there is an interplay of Tet proteins and other epigenetic programs, which may or may not require the oxidative function of Tet proteins to ultimately regulate gene expression. Given this interplay, the further refinement of the combinatorial interaction of different epigenetic programs with Tet proteins will be important to understand how *Tet* gene deletion ultimately regulates gene expression and cell fate decisions.

## Conclusions

Tet proteins are required for proper embryonic development [10, 12, 13] and in ESC by interacting with the pluripotency-associated transcription factor Nanog [29]. While the in vivo roles during early embryogenesis has been well characterized, there remains conflicting literature about the role either individually or combinatorially for the Tet proteins in ESCs. To address this gap, we utilized genomic editing to delete different *Tet* genes individually and in combination. Key findings are that loss of *Tet2* by itself displayed the most significant, quantitative block in differentiation during LIF withdrawal. What is most surprising is that *Tet1*^*−/−*^, DKO, and TKO cells phenocopied each other during differentiation and displayed very similar transcriptome changes both prior and following LIF withdrawal. Consistent with other publications however, we were unable to correlate changes in gene expression with altered DNA methylation. Thus, while transcriptome differences are at best only partially attributable to the DNA demethylase activity of Tet proteins. Nonetheless, the baseline changes in the transcriptome induced by loss of *Tet2* in particular are likely at least partially responsible for the inability of these cells to differentiate. Given the isogenic background we utilized and the use of multiple clones for each genotype, our work represents a valuable resource for investigators interested in pluripotency, epigenetics, and the role of Tet proteins in regulating gene expression.

## Methods

### Generation of Tet knockout and Oct4:IRES:EGFP ESC lines

ESC line used has been described previously [30–32]. Briefly, they are a murine 129/SVj derived in our lab through blastocyst outgrowth, and then adapted to grow under feeder free conditions on gelatin. All lines ESCs were grown on gelatin-coated tissue culture plates in DMEM supplemented with 15% FBS, 2% pen/strep, 1% nucleoside mix, 1% L-glutamine, 1% non-essential amino acids, 10^− 4^ M 2ME, and 10^3^ U LIF. Generation of the Oct4:IRES:EGFP was previously described [32]. To generate the Tet knockout lines, published gRNAs [14] were cloned into pSpCas9(BB)-2A-Puro (PX459) V2.0 (Addgene #62988) and transiently transfected into the Oct4:IRES:EGFP line. Briefly, 1–2 × 10^6^ cells were transfected with 2 μg plasmid (total plasmid DNA in the case of DKO and TKO). 24 h later cells were selected with 2 μg/mL puromycin for 2 days and individual clones isolated. Clones were screened by restriction digest using primers listed in Additional file 1: Table S1 [14]. Restriction testing was done with the following pairs: *Tet1*-SacI, *Tet2*-EcoRV, *Tet3*-XhoI. Indels were confirmed by sequencing and loss of protein was confirmed by Western blot. Two (*Tet2*^−/−^, DKO, TKO) or three (*Tet1*^−/−^, WT) individual clones were used for each experiment. To sequence indels, the same primers used for screening the region were used to amplify the appropriate genomic region, cloned, and a minimum of four individual clones were sequenced to confirm the generation of biallelic indels.

### RNA isolation and RT-qPCR

RNA was isolated using TRIzol (Thermo Fisher 15596026) according to the manufacturer’s instructions. Further purification of RNA was done using the Qiagen RNeasy Mini Kit (Qiagen 7404) and converted to cDNA with the iScript cDNA Synthesis Kit (Bio-Rad 4106228). Approximately 20 ng of cDNA was used for each reaction. Primers used are listed in Additional file 4: Table S4. All primers, unless previously published, were designed using mm9.

### Western blot

Total protein was extracted following lysis in RIPA buffer (25 mM Trizma pH 7.4, 150 mM NaCl, 0.1% SDS, .5% sodium deoxycholate, and 1% NP-40 substitute (Sigma, 74385). Protease inhibitors were added to RIPA as follows: 1:1000 DTT (Sigma, 646563), 5:1000 PMSF (Sigma, 93482-50ML-F), and 1:1000 Protease Inhibitor Cocktail (Sigma, P8340). 10 μg of protein was run for each sample on a 4–20% Criterion Tris-HCl protein gel (Bio-Rad 3450033) and processed using standard western blot technique. All primary incubations were performed overnight in 5% BSA/TBST. Secondary incubations were done in 5% milk/TBST for approximately 1 h. Imaging was performed on a GE Amersham Imager 600. Analysis was done in ImageJ and values were normalized to GAPDH. Western Blot: Sall4 (Abcam, ab29112), GAPDH (Santa Cruz, sc25778), Nanog (Santa Cruz sc8822), Oct3/4 (Santa Cruz, sc9081), Tet1 (Abcam, ab191698), Tet2 (Abcam, ab94580), donkey anti-rabbit IgG-HRP (Santa Cruz, sc2313), m-IgGκ BP-HRP (Santa Cruz, sc516102), Brachyury/T (Santa Cruz, N-19 sc17743).

### LIF withdrawal and flow cytometry

Approximately 5,000 ESC were plated on one well of a six-well gelatin-coated plate in ESC media with LIF and replaced the following day with ESC media lacking LIF. Media was changed every day and on day 6 the cells were analyzed using the BD LSR II flow cytometer and analyzed with FlowJo.

### Statistical analysis

Statistically significant differences were measured using a two-tailed Student’s t-test and a *p*-value <.05. Bar graphs represent the mean of all experiments and errors bars are standard error of the mean (SEM).

### Next-generation sequencing library preparation

#### RNA-seq

1 μg total RNA was obtained, as described above; ERCC RNA (Thermo 4456740) was then added to each sample prior to Poly-A mRNA selection (NEB E7490). RNA-seq libraries were made using the NEBNext Ultra RNA Library Prep Kit for Illumina (E7530). The following numbers of clones were used for both RNA-seq and RRBS analysis: 3 WT, 3 *Tet1*^−/−^, 2 *Tet2*^−/−^, 2 DKO, and 2 TKO. All libraries were run as paired-end (38 × 2, total of 76 cycles) on an Illumina NextSeq 500.

#### RRBS

Libraries were made from 100 ng gDNA using Premium Reduced Representation Bisulfite Sequencing Kit (Diagenode C02030033). All libraries were run as paired-end (38 × 2, total of 76 cycles) on an Illumina NextSeq 500.

### RRBS analysis

Raw sequence reads had the first 6 base pairs clipped off the 5′ end and were also trimmed to remove both poor quality calls using Trim Galore v0.50 (−-clip_r1 6) (www.bioinformatics.babraham.ac.uk/projects/trim_galore/). Adapter sequences were removed with Cutadapt v1.16 [33]. The refined sequences were mapped to mouse reference genome (mm9) using Bismark v0.19.1 [34] with Bowtie2 v2.1.0 [35] at default parameters.

The R package methylKit v1.4.1 [36] was used for further analysis. The aligned files from Bismark were utilized to extract the methylation calls occurring at only CpG dinucleotides with minimum of 10 read coverage. Differentially Methylated Regions (DMRs) were identified for each comparison between treatment and wild type. DMRs were selected based on q-value < 0.05 and those that meet the minimum percent methylation difference cut-off of 25%. Reported DMRs were then annotated using HOMER v4.10 software [37] and the distribution of DMRs in different genomic elements were plotted. Promoters were defined as -1 kb to + 100 bp around TSS of RefSeq gene. Intergenic partitions were defined as genomic regions that did not overlap with promoters, exons and introns.

### RNA-seq analysis

The raw RNA sequence reads were mapped to mouse reference genome build mm9 using STAR v2.5.1 [38] using default parameters and including normalization using the ERCC spike-ins. Quality control matrices were confirmed with a FastQC program. Differential Expression analysis was done using DESeq package in R [39]. Differentially expressed genes were called as significant at Benjamin-Hochberg adjusted *p*-value < 0.05 and fold change of 2. Principal Component Analysis (PCA) was done in R to see the variance between the samples. Heatmaps were done in R using heatmap.2 in R with Pearson method for distance and average agglomeration for clustering. Top 1000 variable genes with log2 read counts were plotted in heatmap after removing duplicates and miRNA genes.

## Additional files

Additional file 1: **Table S1.** Primers and gRNAs. (XLSX 13 kb)

Additional file 2: **Table S2.** RNA-seq readcounts at D0 and D6 for all samples. (XLSX 3202 kb)

Additional file 3: **Table S3.** RRBS DMRs. (XLSX 3901 kb)

Additional file 4: **Table S4.** Indels Generated within all edited lines. Sequencing data for all clones used, indicating the indels induced by genomic editing. Where a single allele is listed, only a single allele was located, but the presence of a large indel which prevents proper PCR amplification of the genomic region cannot be excluded. (DOCX 18 kb)

## Data Availability

All data sets generated in this publication have been submitted to the Gene Expression Omnibus (GEO) under SuperSeries GSE122814.

## Abbreviations

2ME: 2-MercaptoEthanol
5caC: 5-CarboxyCytosine
5fC: 5-FormylCytosine
5hmC: 5-HydroxyMethylCytosine
5mC: 5-MetyhlCytosine
AP: Alkaline Phosphatase
BER: Base Excision Repair
BSA: Bovine Serum Albumin
cDNA: Complementary DNA
CpG: Cytosine-Guanine dinucleotide
D0: Day 0, prior to LIF withdrawal
D6: Day 6 following LIF withdrawal
DKO: Double KnockOut- *Tet1*^*−/−*^*;Tet2*^*−/−*^
DMEM: Dulbecco’s Modified Eagle’s Media
DMR: Differentially Methylated Regions
DNMT: DNA MethylTranferase
DTT: DiThioThreitol
eGFP: Enhanced Green Fluorescent Protein
ESC: Embryonic Stem Cells
FBS: Fetal Bovine Serum
gRNA: Guide RNA
HRP: HorseRadish Peroxidase
ICM: Inner Cell Mass
Indel: Insertion or Deletion
KO: KnockOut of a single gene such as *Tet1*^*−/−*^ or *Tet2*^*−/−*^
LIF: Leukemia Inhibitory Factor
MFI: Mean Fluorescence Intensity
mm9: *Mus musculus* genomic build 9
PCA: Principal Component Analysis
Pen/Strep: Penicillin and Streptomycin
PRC2: Polycomb Repressive Complex 2
RIPA: RadioImmunoPrecipitation Assay Buffer
RNA-seq: Next Generation sequencing of RNA for transcriptomics
RRBS: Reduced Representation Bisulfite Sequencing
RT-qPCR: Reverse Transcription Quantitative PCR
SEM: Standard Error of the Mean
TBST: Tris-Buffered Saline with 0.1% Triton X-100
Tet: Ten Eleven Translocation
TKO: Triple KnockOut- *Tet1*^*−/−*^*;Tet2*^*−/−*^*;Tet3*^*−/−*^
TSS: Transcriptional Start Site
WT: Wild-Type
