# Supplementary material for: Deletion of Tet proteins results in quantitative disparities during ESC differentiation partially attributable to alterations in gene expression
Source: BMC Dev Biol. 2019 Jul 8;19:16. doi: 10.1186/s12861-019-0196-6 (PMC6615237; doi:10.1186/s12861-019-0196-6)
Supplement: Supplementary file 4 — Table S4. Indels Generated within all edited lines. Sequencing data for all clones used, indicating the indels induced by genomic editing. Where a single allele is listed, only a single allele was located, but the presence of a large indel which prevents proper PCR amplification of the genomic region cannot be excluded. (DOCX 18 kb) [file 12861_2019_196_MOESM4_ESM.docx]

**Table 1:** Indels Generated within all edited lines

| **Genotype** | ***Tet1*** **Locus** | ***Tet2* Locus** | ***Tet3* Locus** |
| --- | --- | --- | --- |
| Tet1 KO Clone 1 | Tet1 GTGGCTGCTGTCAGGGAGCTCA**TGG**AGACTAGGTG  Allele 1 ..................---------........  Allele 2 ................----------------... |  |  |
| Tet1 KO Clone 2 | Tet1 GTGGCTGCTGTCAGGGAGCTCA**TGG**AGACTAGGTG  Allele 1 ............------.................  Allele 2 ...............-------............. |  |  |
| Tet1 KO Clone 3 | Tet1 GTGGCTGCTGTCAGGGAGCTCA**TGG**AGACTAGGTG  Allele 1 .............---------.............  Allele 2 .................----------........ |  |  |
| Tet2 KO Clone 1 |  | Tet2 GTGAAAGTGCCAACAGATATCC**AGG**CTGCAGA  Allele 1 ................-------------... |  |
| Tet2 KO Clone 2 |  | Tet2 GTGAAAGTGCCAACAGATATCC**AGG**CTGCAGA  Allele 1 ................--------........ |  |
| DKO  Clone 1 | Tet1 GTGGCTGCTGTCAGGGAGCTCA**TGG**AGACTAGGTG  Allele 1 ..................----------.......  Allele 2 ....------------................... | Tet2 GTGAAAGTGCCAACAGATATCC**AGG**CTGCAGA  Allele 1 ...................-------------  Allele 2 ................................  ^ A Insertion |  |
| DKO  Clone 2 | Tet1 GTGGCTGCTGTCAGGGAGCTCA**TGG**AGACTAGGTG  Allele 1 ..................---------........  Allele 2 ----------------------------------- | Tet2 GTGAAAGTGCCAACAGATATCC**AGG**CTGCAGA  Allele 1 ................--------........  Allele 2 ...C.....A..G...C..--........... |  |
| TKO  Clone 1 | Tet1 GTGGCTGCTGTCAGGGAGCTCA**TGG**AGACTAGGTG  Allele 1 .............T....ACT---------.....  Allele 2 ........------------............... | Tet2 GTGAAAGTGCCAACAGATATCC**AGG**CTGCAGA  Allele 1 ............------------------..  Allele 2 ..................-............. | Tet3 GGCAAGGAGGGGAAGAGTTCTCG**AGG**CTG  Allele 1 ..................--......... |
| TKO  Clone 2 | Tet1 GTGGCTGCTGTCAGGGAGCTCA**TGG**AGACTAGGTG  Allele 1 .................----..............  Allele 2 .................-................. | Tet2 GTGAAAGTGCCAACAGATATCC**AGG**CTGCAGAA  Allele 1 ................--------.........  Allele 2 ..................-.............. | Tet3 GGCAAGGAGGGGAAGAGTTCTCG**AGG**CTG  Allele 1 .........----------------.... |

. Indicates identity with the genomic region (mm9)

- Indicates a deletion

^ Indicates sites of an insertion

Underlined nucleotides are from the gRNA

**Bold** nucleotides indicates the PAM

Where a single allele is listed, we cannot exclude that the second allele contains a large indel which can not be PCR amplified.
